# Marine Bioactive Peptides—Structure, Function and Application

**DOI:** 10.3390/md21050275

**Published:** 2023-04-28

**Authors:** Chang-Feng Chi, Bin Wang

**Affiliations:** 1 National and Provincial Joint Engineering Research Centre for Marine Germplasm Resources Exploration and Utilization, School of Marine Science and Technology, Zhejiang Ocean University, Zhoushan 316022, China; 2 Zhejiang Provincial Engineering Technology Research Center of Marine Biomedical Products, School of Food and Pharmacy, Zhejiang Ocean University, Zhoushan 316022, China

Marine organisms live in harsh marine habitats, causing them to have significantly different and more diverse proteins than those of terrestrial organisms. The unique amino acid sequences hidden in marine proteins can be released via proteolytic hydrolysis and present a variety of biological activities, which provide multiple benefits to human health [1,2,3]. Recently, diverse bioactive peptides have been prepared from marine organisms and their processing byproducts [4,5,6]. Beyond their important nutritional benefits, bioactive peptides showed remarkable bioactivities and pharmacological functions, including antioxidant, anti-inflammatory, cytotoxic, neurotoxic, anti-photoaging, anticoagulant, antidiabetic, antifreeze, endotoxin-binding, lipid-lowering and immune-modulating [1,7,8]. These functions make marine peptides an attractive molecular basis for the design of innovative hepatoprotective drugs, anti-aging drugs, immune-enhancing products, hypolipidemic drugs, anti-photoaging skin care products, etc. [8,9,10]. Therefore, marine-derived bioactive peptides draw great attention to consumers and researchers due to their full possibilities applied in functional foods and medicines.

This Special Issue “Marine Bioactive Peptides—Structure, Function and Application” (https://www.mdpi.com/journal/marinedrugs/special_issues/marine_peptides_structure_function_application, accessed on 3 March 2022) published 10 peer-reviewed research papers on different topics related to marine bioactive peptides, including isolation and structure identification, pharmacological functions and mechanisms and applications in medicines and foods. Herein, we introduce a brief overview of the main achievements contributed by the authors.

Excessive reactive oxygen species (ROS) destroy cell membranes and biomacromolecules in humans, which further trigger a series of chronic diseases. However, the applications of synthetic antioxidants are limited because of their underlying damage [11]. Therefore, looking for potential natural antioxidants from fish-processing byproducts to replace synthetic products has become a research hotspot. At present, polypeptides of *Urechis unicinctus* are mainly extracted from the body’s wall muscle, and the internal organs are directly thrown away as waste, which not only causes environmental pollution but also leads to a waste of resources. Therefore, Li et al. [12] extracted three polypeptides (including VTSALVGPR, IGLGDEGLRR and TKIRNEISDLNER) from the viscera of *U. unicinctus* to improve the utilization value of *U. unicinctus*. Moreover, VTSALVGPR, IGLGDEGLRR and TKIRNEISDLNER can concentration-dependently protect RAW264.7 cells against H_2_O_2_-induced oxidative damage. This research suggested that VTSALVGPR, IGLGDEGLRR and TKIRNEISDLNER might serve as potential antioxidants applied in health-derived food or beverages. In addition, this study further developed a new use for the byproduct of *U. unicinctus*, which improved the comprehensive utilization of marine biological resources.

Non-alcoholic fatty liver disease (NAFLD) refers to a clinicopathological syndrome characterized by inflammation of the liver lobule and hepatic parenchymal steatosis [13]. Lipid accumulation (or lipotoxicity), oxidative stress and inflammation exert critical impacts in the development of NAFLD [14]. Ye et al. [15] reported that monkfish peptides could ameliorate high-fat diet (HFD)-induced NAFLD in mice, and the mechanism demonstrated that monkfish (*Lophius litulon*) peptides can first improve the lipid metabolism in NAFLD mice via an upregulation of p-AMPK protein to reduce lipid synthesis and accelerate fatty acid β-oxidation. Secondly, monkfish peptides showed strong antioxidant activity to decrease oxidative damage by regulating the Nrf2 pathway to increase HO-1 and NQO1 levels in mouse livers. Finally, monkfish peptides can reduce the levels of inflammatory factors (TNF-α, IL-1β, IL-6 and IFN-γ). Therefore, monkfish peptides can be used as health functions or supplements to prevent and treat NAFLD.

Sturgeon is the common name for 27 kinds of cartilaginous fish of the family Acipenseridae, and its farmed production in China was about 4.4 million tons, accounting for approximately 80% of the world’s production [16,17]. Cartilages accounting for 10% of sturgeon’s weight became byproducts during the receiving process of sturgeon eggs. In recent years, bioactive ingredients in sturgeon cartilage were studied constantly for replacing shark cartilage used in health and functional products. Therefore, Sheng et al. [18] isolated and identified thirteen antioxidant peptides from the collagen hydrolysate of Siberian sturgeon (*Acipenser baerii*) cartilage, and GEYGFE, PSVSLT and IELFPGLP showed high antioxidant activity and protective capacity on H_2_O_2_-damaged plasmid DNA. More importantly, GEYGFE, PSVSLT and IELFPGLP displayed notable cytoprotection on vascular endothelial cells against H_2_O_2_ injury by regulating the intracellular antioxidant system to decrease the contents of ROS and malondialdehyde (MDA). Therefore, this research provides technical support for the higher-valued utilization of sturgeon cartilages. More importantly, GEYGFE, PSVSLT and IELFPGLP might act as antioxidant additives for developing health products to treat chronic diseases caused by oxidative stress, including cardiovascular, atherosclerosis, hypertension, etc.

O/W emulsion is a classic form of oil commonly used in foods, but it is easily oxidized. Some antioxidative proteins and hydrolysates can be used as emulsifiers and antioxidants in different types of oils to increase the nutritional value of emulsions. Therefore, Liu et al. [19] prepared the neutral protease chlorella protein hydrolysate (NCPH) and the alkaline protease chlorella protein hydrolysate (ACPH) and systematically studied their antioxidant activities and physicochemical properties in krill oil-in-water emulsions. NCPHs and ACPHs could significantly increase SOD activity to reduce H_2_O_2_-induced oxidative stress on MDA-MB-231 cells in a dose-dependent manner. Moreover, the NCPHs or ACPHs could inhibit linoleic acid oxidation, suppress the growth of the peroxide value (POV) and thiobarbituric acid reactive substances (TRABS) and remain physically stable in krill O/W emulsion for at least one month. The study suggests that protein hydrolysate from chlorella pyrenoidosa has the potential to be applied in krill oil-in-water emulsions, as both emulsifiers and antioxidants.

Wound healing involves three stages: inflammation, cell proliferation and tissue reconstruction [20]. The three phases of this lengthy procedure can involve a number of symptoms, including infection, discomfort and the development of thicker scars. To make matters worse, slow wound healing not only affects the patient’s aesthetics but also seriously affects the patient’s quality of life. Therefore, screening original drugs with functions of speeding up skin wound closure and inhibiting scar formation are essential for patients with tissue defects. In this regard, Zhang et al. [21] prepared the best fraction PMPs-4 (PMPPs) from the ultrafiltration fraction (<3 K Da) of shellfish *Pinctada martensii* mantle (PMPs) via gel chromatography. Cellular assays proved that PMPPs could promote the proliferation of HSF and HaCaT cells, and in vivo animal experiments indicated that PMPPs could achieve scarless healing by regulating the TGF-β/Smad pathway to inhibit the inflammatory response, accelerate the epithelialization process and regulate the collagen I/III ratio. Finally, the most promising peptide sequence FAFQAEIAQLMS in PMPPs can promote wound healing through easy docking with protein receptors of EGFR1/FGFR1/MPP-1. This research enriches the pro-healing mechanism of marine peptides and provides candidate drugs for treating wound healing.

Various internal and external factors, such as viral infections, cancer, obesity, smoking, excessive alcohol consumption, and very little exercise, show negative effects on the normal functioning of the immune system. Immunotherapy can improve or suppress the body’s immunological responses for returning the immune system to its physiologic status. In previous research, a novel immune-enhancing pentadecapeptide (RVAPEEHPVEGRYLV) was isolated from protein hydrolysates of the bivalve *Cyclina sinensis*, and it showed significant immunomodulatory effects on RAW264.7 cells and an ameliorating effect on cyclophosphamide-induced nephrotoxicity [22,23]. In the study, Zhao et al. [24] found that RVAPEEHPVEGRYLV could enhance immunity in CTX-induced immunosuppressed mice, and the mechanism indicated that RVAPEEHPVEGRYLV could activate the MAPK/NF-κB and PI3K/Akt pathways to elevate the phosphorylation levels of p38, ERK, JNK, PI3K and Akt, upregulate IKKα, IKKβ, p50 NF-κB and p65 NF-κB protein levels and downregulate IκBα protein levels. The study supports RVAPEEHPVEGRYL serving as a novel immunomodulator candidate or immune adjuvant.

Hypertension seriously affects the morbidity and mortality of cardiovascular and renal diseases. Angiotensin (Ang) I-converting enzyme (ACE) is the key protease in participating in the regulation of blood pressure through the renin–angiotensin system, and inhibiting ACE activity is an ideal method in hypertension treatment [25]. The consumption of red algae is actually expanding because they are a good source of essential nutrients, minerals and vitamins, and they also contain bioactive compounds with attractive biological activities. In this study, Mune et al. [26] used thermolysin to hydrolyze water-soluble proteins of red alga *Gracilariopsis chorda* to prepare a hydrolysate with high ACE inhibitory activity. Furthermore, two novel peptides (IDHY and LVVER) were isolated from the prepared hydrolysate, and molecular docking analysis revealed that IDHY was a promising ACE inhibitor. From these results, it could be expected that a water-soluble protein hydrolysate of *G. chorda* could serve as an ingredient in the prevention of hypertension and a good source of bioactive peptides, especially ACE inhibitory peptides.

Brown algae *Laminaria digitata* contains a variety of functional components with significant pharmacological activity, such as alginate, fucoxanthin, fucoidan, phlorotannins and vitamins. The mechanism of the antihypertensive effect of bioactive peptides is thought to inhibit enzyme activity within the renin–angiotensin–aldosterone system (RAAS), including ACE-1 and renin. In silico analysis is a useful technique to speculate on the potential bioactivity of peptides before the chemical synthesis of peptides. Previously, Purcell et al. [27] generated a protein hydrolysate of *L. digitata* with an IC_50_ value of 590 μg/mL on ACE-1. In this paper [28], 130 peptides were identified from the 3 kDa permeate of this hydrolysate using mass spectrometry. Among them, two new ACE-1 inhibitory peptides of IGNNPAKGGLF and YIGNNPAKGGLF with Peptide Ranker scores of 0.81 and 0.80 were chemically synthesized and inhibited ACE-1 by 80 ± 8% and 91 ± 16%, respectively. The observed ACE-1 IC_50_ values for IGNNPAKGGLF and YIGNNPAKGGLF were determined as 174.4 µg/mL and 133.1 µg/mL. What is interesting is that IGNNPAKGGLF and YIGNNPAKGGLF have the potential to inhibit dipeptidyl peptidase IV (DPP-IV) by mimicking sequences produced after digestion. The study highlights that *L. digitata* is an excellent material for preparing peptides with ACE and DPP-IV inhibitory activity.

Prolonged skin exposure to Ultraviolet B (UVB) radiation can result in detrimental intracellular physiological effects and produce superfluous ROS, which can injure intracellular bioactive molecules and further cause oxidative stress and cell apoptosis [29]. Then, inhibiting the photoaging induced by UVB can delay skin aging and provide a reasonable basis for studying cosmetic products to treat diseases caused by UV radiation. Recently, bioactive peptides from marine organisms have exhibited great possibilities for the adjuvant treatment and prevention of skin photoaging due to their outstanding antioxidant function. In previous research, PKK, YEGGD and GPGLM from skipjack tuna cardiac arterial bulbs were found to have excellent radical scavenging ability and a prominently protective function on H_2_O_2_-damaged DNA and HepG2 cells [30]. Therefore, this paper discussed the protective effects of PKK, YEGGD and GPGLM on the cells damaged by UVB oxidation from two aspects: antioxidant and apoptosis inhibition [31]. The results indicated that PKK, YEGGD and GPGLM could significantly increase the cellular antioxidant capacity through activating the Nrf2 signaling pathway and suppressing cell apoptosis through downregulating Bax-dependent mitochondrial apoptosis. This work lays a theoretical foundation for employing PKK, YEGGD and GPGLM to attenuate UVB-irradiated photoaging.

Oxidative stress can cause DNA mutation, enzyme inactivation and membrane phospholipid oxidation, which significantly increase the incidence of some chronic non-communicable diseases, such as neurological dysfunction, hypertension, cardiovascular, atherosclerosis, diabetes, cancer and auto-immune diseases [32,33,34]. Sheng et al. [35] utilized processing byproduct swim bladders of monkfish (*L. litulon*) to produce eighteen novel antioxidant peptides, and YDYD, QDYD, GRW, ARW, DDGGK and YPAGP revealed remarkable radical scavenging activity, lipid peroxidation inhibition ability, ferric-reducing antioxidant power and protective function on oxidation-damaged DNA and HepG2 cells. Furthermore, the stability of eighteen novel antioxidant peptides is systematically discussed. This research provides a good perspective and offers technical support for the higher-valued utilization of monkfish byproducts. More importantly, YDYD, QDYD, GRW, ARW, DDGGK and YPAGP might act as antioxidant additives for developing health products to cure chronic non-communicable diseases.

The papers included in this Special Issue deal with bioactive peptides from evolutionarily distant species, including *U. unicinctus*, monkfish (*L. litulon*), Siberian sturgeon (*A. baerii*), *C. Pyrenoidosa*, *P. martensii*, *C. sinensis*, *G. chorda*, *L. digitate* and skipjack tuna (*K. pelamis*). Among them, three of the papers involved the preparation of bioactive peptides from seaweed. These results suggest that seaweed is not only an important raw material for the preparation of polysaccharides but also a potential resource for the preparation of peptides. This should be a phenomenon of concern. Moreover, most of the identified peptides displayed broad-spectrum biological activities. To our surprise, the papers included in this Special Issue pay more attention to the functions and mechanisms of peptides in addition to the conventional isolation and identification of peptides. This means that more marine bioactive peptides will be used in pharmaceutical, cosmeceutical and nutraceutical industries in the future.

In conclusion, the Guest Editors thank all the authors who contributed to this Special Issue, all the reviewers for evaluating the submitted manuscripts and the Editorial board of *Marine Drugs*, especially Florine Wang, Assistant Editor of this journal, for their continuous help in turning this Special Issue into a reality.
